# Masticating Stress

**Published:** 1900-10

**Authors:** B. F. Arrington

**Affiliations:** Goldsboro, N. C.


### MASTICATING STRESS.

BY B. F. ARRINGTON, D.D.S., GOLDSBORO, N. C.

THE *Dental Cosmos* for June, 1895, contains a paper written by Dr. G. V. Black, in which he says, "In the *Dental Cosmos* for May I have given in some detail studies of the physical character of filling-materials. It is necessary to determine as nearly as possible the stress to which they will be subjected in the human mouth. This requires of us the determination of the force with which the teeth may be brought in occlusion by voluntary effort, and the stress usually employed in eating or in the usual mastication of the different articles of food. These determinations seem necessary as a basis for decision as to the strength required of materials to prevent them from being crushed or dislodged by the stress of mastication when placed in the teeth as fillings."

Then the doctor discourses at length upon the subject of force requisite in the ordinary mastication of food and sundry articles, and proves conclusively, he seems to think, by the use of his *phagodynamometer,* that his statements are correct and must stand as truth established on a scientific basis and by strictly scientific tests performed in the presence of men of prominence in the dental profession.

His tabulated statement of masticating force requisite for crushing various articles named, with one exception, varies from fifteen to one hundred and seventy-three pounds for each occlusion of the jaws. The statement and exhibit he makes are most unreasonable and extreme, and have no feature of strength rightly based to sustain them, and can be easily proved erroneous and misleading by the use of a simple and much more practical contrivance, easily improvised for testing strength and endurance of the muscles called into action in the exercise of masticating food, than the phagodynamometer, to which Dr. Black and others attach so much importance.

That the little instrument so favorably endorsed by Dr. Black will crush meats and crack nuts and candies under the stress of *lever power* as applied by Dr. Black, no one will doubt, but to sanction his theory and claim, that it measures and marks correctly in pounds the stress of crushing force requisite in the ordinary process of mastication, would be an unreasonable stretch of conviction, and it is asking too much of the dental profession to accept any such theory, unless better sustained by features and facts based upon practical as well as *scientific* principles, that tests made and results obtained may stand as truth and strengthen with time.

Dr. Black will of necessity have to devise and produce a better testing-machine, one more trustworthy and correct in its workings, before he can reasonably hope to prove to the profession that amalgam fillings in cavities will *flow* and *spread* under *ordinary masticating stress,* or any other order of stress possible to be applied to fillings in teeth.

The *flow* theory is weak, very weak, and it will be difficult ever to strengthen and sustain it, from the fact that there is no reasonable and secure foundation in fact to build upon. Sometimes, in attempting to carry a point in effort to strengthen a weak theory, the theory is more weakened and the sooner proves a failure, as in this instance of *stress and flow* advocated and championed by Dr. Black. Extremes and false theory never operate for good in any cause. Emanate from what source they may, they never savor of good. Let them go and go quickly if possible; the sooner out of sight and forgotten the better.

All will admit and none question the extent of possibilities of the extreme force that can be temporarily exerted in the exercise of the occlusion of the jaws in biting or cracking hard substances, but to say amen to Dr. Black's teaching pertaining to the subject, and endorse his tabulated exhibit of stress to sustain his theory of "amalgam flow," would be a stultification of independent thought, judgment, and the right to investigate. It would prove a hinderance to true progress in professional work, and would greatly tend to establish a hurtful precedent, a willing acceptance of false theories.

The doctor, in his scientific efforts to establish and sustain his point that amalgam fillings *flow* under *masticating stress,* seems to have lost sight of the fact that he imposes an unreasonable and unbearable tax upon the peridental membrane and the few muscles called into action in the occlusion of the jaws, to say nothing of the teeth, a tax far greater than the possibility of membrane and muscular endurance, too extreme and impractical to be favorably entertained and considered as a possibility for a single moment.

To make the subject brief, I will mention and comment upon three articles only enumerated in the doctor's statement of experimental tests as recorded, pages 483 and 484. He says, "In the ordinary or usual process of mastication, beef-steak well done and rather tough requires a stress varying from sixty to eighty pounds, lemon-drops from sixty to seventy-five pounds, and hazel-nuts, old and dry, from one hundred and fifteen to one hundred and seventy-three pounds." Eight were tested, and made an average of *one hundred and forty-seven pounds.* Just think, and for each occlusion of the jaws! Strong muscles, certainly.

As regards beef-steak, it will not require argument or effort on any line to convince any one at all familiar with children and their accustomed habits and indulgences at table during meals that the majority of them at the age of seven or eight years, with only four permanent molars and temporary teeth, possibly defective, will masticate beef-steak easily and quickly and without apparent effort. The occlusion of the jaws during a single meal will possibly range from one hundred and fifty to two hundred and fifty, a reasonable estimate; for average, say, two hundred occlusions during a meal and *seventy pounds* stress for each occlusion, according to Dr. Black's showing the result would be fourteen thousand pounds,—out of all reason and contrary to sound judgment and facts, and cannot be established and sustained by science ordinary or extraordinary, with the aid of the phagodynamometer or any other machine yet invented. Such a strain and tax upon the anatomy involved during the exercise of eating a meal, a period of fifteen or twenty minutes, about the usual time consumed by children at table eating a square meal, embracing beef-steak, would soon prove hurtful and destructive beyond possibility of repair and restoration to a normal state. Couple with this unreasonable and extravagant feature the fact that not one child in fifty of mentioned age is equal to the task of lifting and shouldering a weight of sixty pounds, the minimum claimed by Dr. Black as requisite in occlusal stress in eating steak. With the whole muscular exertion and strength of body put forth, such a feat at such an age could not be performed.

I will ask, and Dr. Black can explain possibly, how it is possible for the few muscles involved to undergo such strain and affect the extreme stress he represents to be common to all? In my judgment there can be but one fair and honest answer given that is reasonable and will be acceptable, and that is, that beautiful little piece of scientific, mechanical ingenuity, the phagodynamometer is in fault.

It has played deceptive and fooled many, consequently should be investigated and carefully looked into and made to do better work, and stop misleading the easy-to-be-led.

In reference to hazel-nuts and lemon-drops, said to require such extreme muscular force and strength of jaws for cracking and crushing, I will relate results of some recent experiments on that line, which possibly may interest some. I procured of a confectioner hazel-nuts and lemon-drops, and found a boy about eleven years old who was willing for a small compensation to exercise his jaws as required. The boy was of average build and weight, with teeth that might be classed fair average. He commenced with the hazel-nuts, placing one at a time between the molars on the right side, and continued cracking and removing from his mouth, without cessation, until he had cracked one hundred and fifty, and in less time than six minutes. Then, without waiting, proceeded to crack (one at a time) the same number of lemon-drops, and in about the same space of time, altogether less than twelve minutes, and without perceptible fatigue or strain of muscles in evidence. The boy expressed satisfaction and willingness to repeat the performance immediately if desired. Estimating according to Dr. Black's tabulated statement of stress requisite for cracking hazel-nuts and lemon-drops, the average foots up as follows: One hundred and forty-nine pounds each for hazel-nuts and sixty-five pounds each for lemon-drops; total thirty-one thousand eight hundred pounds; as would be claimed and proclaimed by the converts and advocates of the phagodynamometer. But there was no such stress applied in cracking of nuts and lemon-drops by the eleven-year-old boy, let the machine talk as it may. As perfect as it may be in mechanism, it is not suited to determine correctly the occlusal stress of muscles in eating. It is due to truth, to science, and to deceived and misled dentists to so state. Time and investigation will confirm and sustain the correctness of my statements. I must state that the boy who cracked the nuts and lemon-drops was put to a test for exhibit of physical strength, and with all the muscular power at his command failed to lift a weight of seventy-five pounds, a weight less by seventy-two pounds than the stress required for cracking a single nut as measured by the phagodynamometer, which has during the past few years caused so much talk and played such a conspicuous part in deluding and leading astray many good men in dentistry.

It shows how easily disposed and willing we are to accept a new theory, said to be scientific, when intelligently and pleasantly presented, as Dr. Black is competent to do, and without questioning or testing as best we can to determine whether it is sound or unsound. All men are liable to err, and "all are fallible." I must do Dr. Black the justice to say I believe he would as willingly and quickly correct an error detected in his scientific work as any man living, and that is all that can be required of him as a scientist and an honest dentist, but we hope that wherein he has erred he will not be "forever and a day" finding it out.

Scientific work in dentistry must not be ignored; it is useful and much needed; but we want that it shall be truly scientific and conform to the practical, and harmonize on a basis of good sound judgment, reason, and common sense, that facts when established may be relied upon.

We will hope the time is near at hand when Dr. Black will realize the necessity for revising his experiments and testings, and will so correct, pertaining to masticating stress, as to make his scientific labors on that line perfectly reliable and more universally acceptable than at present, and will openly and frankly say to the profession that he fully realizes wherein he has erred, and, like others, has been misled by that unreliable little pet of his, the phagodynamometer, and that he is now satisfied that, after all his efforts to prove otherwise, it is not so essential that amalgam shall be so extremely hard to resist *masticating stress,* as *flow* of amalgam fillings under ordinary masticating stress is really not within the range of possibilities, viewed from a reasonable practical stand-point (the best point that can be made), especially as it is reasonable to presume that in the absence of the phagodynamometer the occlusal force in masticating ordinarily will not require a stress exceeding a few pounds.

In the event of Dr. Black failing (and fail he must) to sustain his theory pertaining to masticating stress, wherein is there a feature of profitable, practical benefit to dentists in all that he has written and figured on the subject to establish his theory of the extreme stress of mastication and consequent *flow* of amalgam fillings under such stress?

It is sad to think of the loss of so much valuable time and talent that might have been more profitably appropriated. But such is life, and we take the chances.

Dr. Black says, "Dentists have not been in the habit of thinking of the force of mastication as so much in pounds, and when the tests are made and the facts are stated in print many hesitate to believe them, because they have a sentiment that the figure is too high."

A little more sentiment than the doctor has practised would possibly have made his scientific work more effective and reliable. Dr. Black's theory is based on scientific laboratory work, so claimed. What I have said, and the experiments stated, may be based on sentiment. I will ask that any or all interested on the subject test both carefully, with a close study of the anatomy involved, and experiment for results to determine which is practical and which is truth, and decide for themselves if in their judgment and experience there is truly any such thing as flow of amalgam fillings under masticating stress.

To get at truth rightly, especially when confronted by such an ingenious story-telling little machine as the phagodynamometer, we are compelled to experiment a little now and then; otherwise where might we not drift, and what of the consequences?

